# Supplementary material for: The evolution, diversity, and host associations of rhabdoviruses
Source: Virus Evol. 2015 Nov 13;1(1):vev014. doi: 10.1093/ve/vev014 (PMC5014481; doi:10.1093/ve/vev014)
Supplement: Supplementary Data S1 [file ve_vev014_index.html]

Supplementary Data | Virus Evolution

## Supplementary Data

files

- Supplementary Data - xlsx file
- Supplementary Data - xlsx file
- Supplementary Data - docx file
- Supplementary Data - docx file
- Supplementary Data - pdf file
