# Supplementary material for: The evolution, diversity, and host associations of rhabdoviruses
Source: Virus Evol. 2015 Nov 13;1(1):vev014. doi: 10.1093/ve/vev014 (PMC5014481; doi:10.1093/ve/vev014)
Supplement: Supplementary Data S1 [file Supp_tables.docx]

**Table S1: Newly discovered rhabdoviruses (7) from RNA-seq data**

| **Name** | **Abbreviation** | **Host isolated from** | **Location/date** | **Accession** | **Number of genes** | **Contig size** |
| --- | --- | --- | --- | --- | --- | --- |
| Drosophila subobscura rhabdovirus | DSubRV | Drosophila subobscura | Falmouth, UK (2008) | KR822817 | L gene | 7008 |
| Drosophila sturtevanti sigma virus | DStuSV | Drosophila sturtevanti | Granada, Spain (2009) | KR822816 | 5 core genes + X gene between P-M | 13885 |
| Drosophila sturtevanti rhabdovirus | DStuRV | Drosophila sturtevanti | Granada, Spain (2009) | KR822823 | L gene only | 7658 |
| Drosophila busckii rhabdovirus | DBusRV | Drosophila busckii | Ithaca, NY state, USA (2010) | KR822813 | 5 core genes + additional gene between G-L | 14298 |
| Scaptodrosophila deflexa sigma virus | SDefSV | Scaptodrosophila deflexa | Chambon, France (2012) | KR822820  (also see KR822821 + KR822822) | L gene partial sequence only | 997 |
| Drosophila montana sigma virus | DMonSV | Drosophila montana | Oulanka, Finland (2008) | KR822815 | L gene partial sequence only | 5880 |
| Drosophila affinis/athabasca sigma virus | - | Drosophila affinis/athabasca | Ithaca, NY state, USA (2010) | KR822824 | L gene partial sequence only | 7016 |

**Table S2: Viruses with extended sequence (4) from RNA-seq**

| **Name** | **Abbreviation** | **Host isolated from** | **Location/date** | **Accession** | **Number of genes** | **Contig size** |
| --- | --- | --- | --- | --- | --- | --- |
| Drosophila ananassae sigma virus | DAnaSV | Drosophila ananassae | Kilifi, Kenya (2010) | KR822812 | 5 core genes + X gene between P-M | 12791 |
| Drosophila affinis sigma virus (isolate 10A) | DAffSV | Drosophila affinis | Raleigh, NC, USA (2008) | KR822811 | 5 core genes + X gene between P-M | 14448 |
| Drosophila immigrans sigma virus | DImmSV | Drosophila immigrans | Pool from Edinburgh and Sussex, UK (2011) | KR822814 | 5 core genes + X gene between P-M | 12482 |
| Drosophila tristis sigma virus | DTriSV | Drosophila tristis | Pool from Edinburgh, UK (2011) | KR822818 | L gene partial sequence only | 5347 |

**Table 3: TSA database viruses (19)**

| **Name** | **Host isolated from** | **Location/date** | **Accession** | **Contig size** |
| --- | --- | --- | --- | --- |
| Agave tequilana | Agave tequilana | USA (2013) | GAHU01042870.1 | 13148 |
| Lotus corniculatus | Lotus corniculatus | China (2013) | GACB01036222.1 | 5361 |
| Medicago sativa | Medicago sativa (alfalfa) | China (2014) | GAFF01090372.1 | 9111 |
| Oropsylla silantiewi | Oropsylla silantiewi (Himalayan marmot flea) | China (2014) | GAWY01012238.1 | 6872 |
| Caligus rogercresseyi 11125273 | Caligus rogercresseyi (sea louse) | Chile (2014) | GAZX01041484.1 | 7304 |
| Caligus rogercresseyi 11114047 | Caligus rogercresseyi (sea louse) | Chile (2014) | GAZX01030328.1 | 6209 |
| Pararge aegeria rhabdovirus | Pararge aegeria | Belgium (2013) | KR822826 | 13062 |
| Ceratitis capitata sigma virus | Ceratitis capitata | Hawaiian populations and Vienna 7 lab line (2014) | KR822825 | 12583 |
| Spodoptera exigua rhabdovirus | Spodoptera exigua. Probable EVE – only small (~250 AA ORF) | Lab population (2014) | GARL01090000.1 | 3499 |
| Musca domestica virus 1 | Musca domestica (house fly) | China (2014) | GBAB01012379.1  Note high homology to Wuhuan house fly virus 2 | 11329 |
| Humulus lupulus TSA | Humulus lupulus (hops plant) | Japan (2015) | dbj\|LA356888.1 | 4166 |
| Conwentzia psociformis TSA | Conwentzia psociformis (Lacewings) | Germany (2011) | GAYH01091085.1 (also see contigs GAYH01090244.1 + GAYH01089761.1) | 2853 |
| Frankliniella occidentalis TSA | Frankliniella occidentalis (Western flower thrip) | Hawaii (2014) | GAXD01021508.1 | 6630 |
| Triodia sylvina TSA | Triodia sylvina (Orange swift moth) | Germany (2011) | GAVB01022634.1 | 13264 |
| Kerria lacca TSA | Kerria lacca (scale insect) | India (2013) | GBDY01035336.1 | 1419 |
| Corydalus cornutus TSA | Corydalus cornutus (Eastern dobsonfly) | USA (2011) | GATG01003324.1 | 6748 |
| Planococcus citri TSA | Planococcus citri (Citrus mealybug) | Lab stock, Germany (2011) | GAXF01123343.1 | 1814 |
| Lolium perenne TSA | Lolium perenne (Rye grass) | Lab line (2014) | GAYX01123773.1 | 1383 |
| Hydra magnipapillata trimmed TSA* | Hydra magnipapillata | Unknown (2013) | GAOL01026175.1 | 19583 |

*Atypically long L gene (RDRP) or sequencing error- only first 1500 AAs used for phylogeny inference

**Table S4: Rhabdovirus-like sequences (8) with short coding regions**

| **Name** | **Isolated from** | **Blast homology**  **(tblastx top hit)** | **Accession** | **Contig Length** |
| --- | --- | --- | --- | --- |
| Euglossa cordata TSA | Euglossine bee | Taastrup virus | HP981634.1 | 1061 |
| Mengenilla moldrzyki TSA | Strepsiptera | Spring viraemia of carp | JP079120.1 | 628 |
| Chilo suppressalis TSA | Asiatic rice borer (Lepidoptera) | Tacheng Tick Virus 6 | GAJS01034243.1 | 728 |
| Ostrinia nubilalis TSA | European corn borer (Lepidoptera) | Pike fry rhabdovirus | GAVD01036510.1 | 637 |
| Chrysopa pallens TSA | Green lacewing | Tacheng Tick virus 6 | GAGF01049798.1 | 954 |
| Aposthonia japonica TSA | Websppiner | Yug Bogdanovac virus | GAWU01251223.1 | 849 |
| Aedes albopictus TSA | Mosquitoes | Beaumont virus | JO897900.1 | 817 |
| Hydroptila sp. EVE | Microcaddisflies | Muscina stabulans sigma virus/Vesicular stomatitis virus New Jersey/Muscina stabulans sigma virus | GAVM01002013.1/  GAVM01077366.1/  GAVM01002014.1 | 3924/  1365/  3816 |

**Table S5: other mononegavirales (10) from RNA-seq or database searches**

| **Sequence** | Isolated from | Accession | Contig length |
| --- | --- | --- | --- |
| Drosophila unispina virus 1 (RNAseq) | Drosophila unispina from Chambon, France (2012) | KR822819 | 13559 |
| Echinogammarus veneris virus 1 TSA | Echinogammarus veneris | GARO01006190.1 | 2072 |
| Sclerotinia homoeocarpa TSA | Sclerotinia homoeocarpa (dollar spot fungus | JW826636.1 | 8645 |
| Meligethes aeneus TSA | Meligethes aeneus (pollen beetle) | GAPE01009923.1 | 2370 |
| Ganaspis sp G1 TSA | Ganaspis sp Figitid larval parasitoid of Drosophila | GAIW01024762.1 | 4587 |
| Bemisia tabaci TSA | Silverlead whitefly | GARQ01013962.1 | 12158 |
| Stylops melittae TSA | Strepsiptera, that parasitize various species of sand bees | GAZM01009928.1 | 14692 |
| Cordulegaster boltonii TSA | Golden ringed dragonfly | GAYO01003118.1 | 11249 |
| Trialeurodes vaporariorum TSA | Greenhouse whitefly | GAWX01022059.1 | 13816 |
| Bombyx mori EVE TSA | Silk worm | GBJR01003404.1 | 3972 |
