# Supplementary material for: The evolution, diversity, and host associations of rhabdoviruses
Source: Virus Evol. 2015 Nov 13;1(1):vev014. doi: 10.1093/ve/vev014 (PMC5014481; doi:10.1093/ve/vev014)
Supplement: Supplementary Data S1 [file Supplementary_materials.docx]

**Supplementary materials**

Tables S1-5. List of newly discovered viruses:

Figures S1-S4

Data S1, sample information

Data S2, virus ID, Genbank accession numbers and host information

Supplementary Figure 1.

Results of permutation tests of population differentiation between the sequences of viruses taken from different categories of host: (a) between arthropods and vertebrates, (b) between arthropods and plants and (c) between plants and vertebrates. Red lines show the degree of differentiation, measured using Hudson’s *F_st_* estimator, and grey histograms show the *F_st_* values from 1000 unique random permutations of host categories over viral sequences. We found significant differentiation between each pair of categories (*F_st_* values: a=0.07, b=0.35, c=0.48) with none of the 1000 permutations resulting in as high an *F_st_* value as the data (*P*<0.001 for all).

Supplementary Figure 2.

Correlation of the evolutionary distances between rhabdovirueses and the evolutionary distances between their arthropod hosts. Figure shows the results of a permutation test of this relationship (measured by Pearson’s correlation coefficient). The red line shows the true correlation coefficient (0.36), and the grey histogram shows the correlation coefficients returned from 1000 unique random permutations of host genera over viral sequences (see Methods). We found a significant association between viral and host phylogenies with none of the 1000 permutations resulting in as strong a correlation as the data (*P*<0.001).

Supplementary Figure 3.

Results of the permutation test of population differentiation between the sequences of viruses taken from land and aquatic arthropod and vertebrate hosts. Only arthropod and vertebrate hosts were included in the test, since aquatic hosts only included arthropods and vertebrates. The red line shows the true degree of differentiation, measured using Hudson’s *F_st_* estimator, and the grey histogram shows the *F_st_* values returned from 1000 unique random permutations of host categories over viral sequences. Viruses from terrestrial and aquatic hosts were found to have significant population differentiation (with only 7 of the 1000 permutations resulted in as high an *F_st_* value as the data (*F_st_* =0.07, *P*=0.007).

Supplementary Figure 4.

Bayesian maximum clade credibility tree from host association analysis. Branch labels show reconstructed host associations.

**All data has been made available in public repositories:**

NCBI Sequence Read Archive Data: SRP057824

Results from testing ancestral trait reconstructions predictions: http://dx.doi.org/10.6084/m9.figshare.1538584

L gene sequences fasta: <http://dx.doi.org/10.6084/m9.figshare.1425067>

TrimAl alignment fasta: <http://dx.doi.org/10.6084/m9.figshare.1425069>

Gblocks alignment fasta: <http://dx.doi.org/10.6084/m9.figshare.1425068>

Phylogenetic tree Gblocks alignment: <http://dx.doi.org/10.6084/m9.figshare.1425083> Phylogenetic tree TrimAl alignment: <http://dx.doi.org/10.6084/m9.figshare.1425082>

BEAST alignment fasta: <http://dx.doi.org/10.6084/m9.figshare.1425431>

BEAUti xml file: <http://dx.doi.org/10.6084/m9.figshare.1431922>

Bayesian analysis tree: <http://dx.doi.org/10.6084/m9.figshare.1425436>
