# Supplementary figures and images for: The evolution, diversity, and host associations of rhabdoviruses
Source: Virus Evol. 2015 Nov 13;1(1):vev014. doi: 10.1093/ve/vev014 (PMC5014481; doi:10.1093/ve/vev014)

Supplementary Figure 1

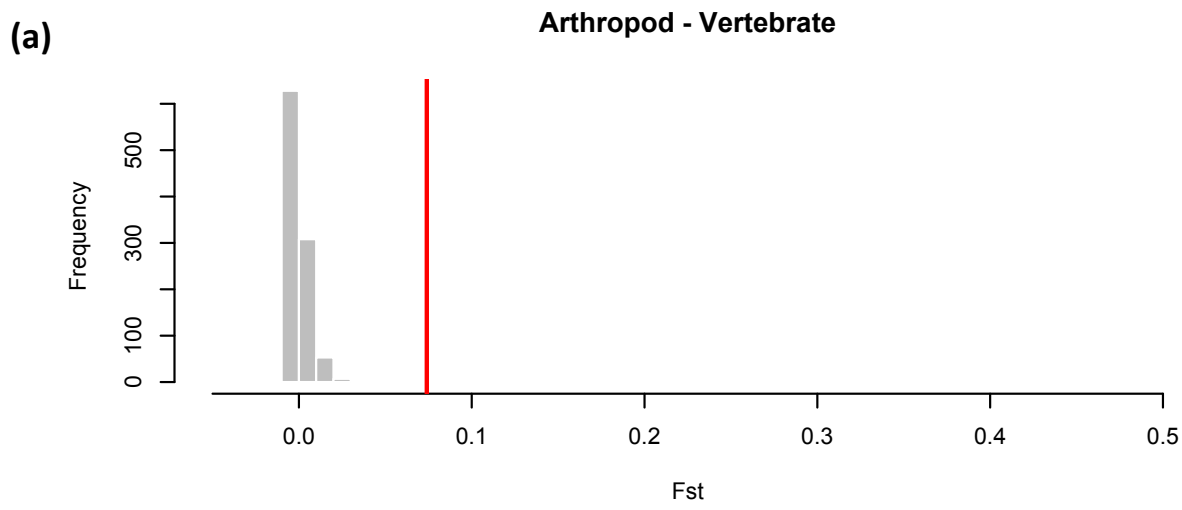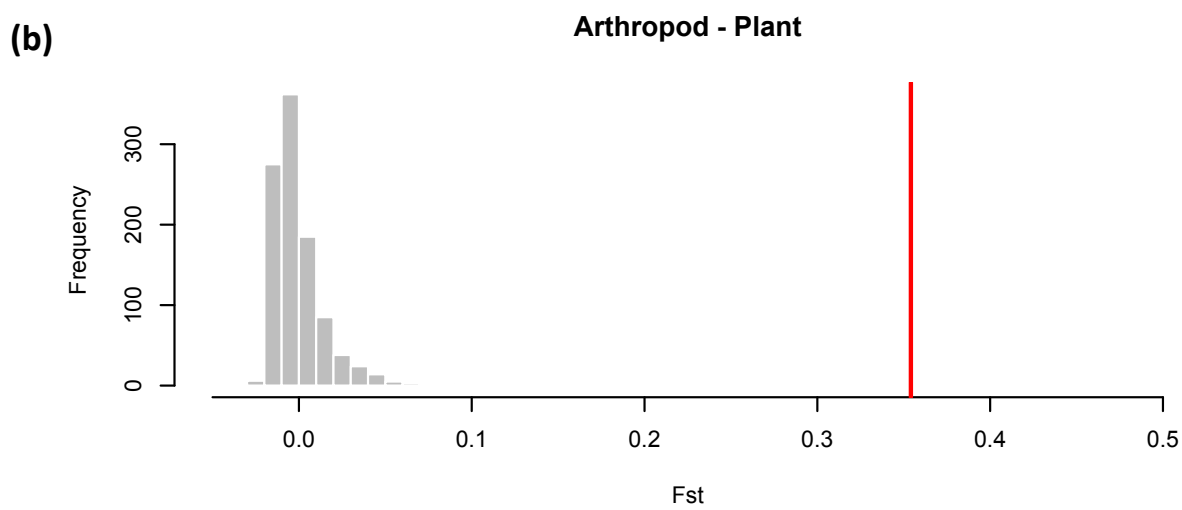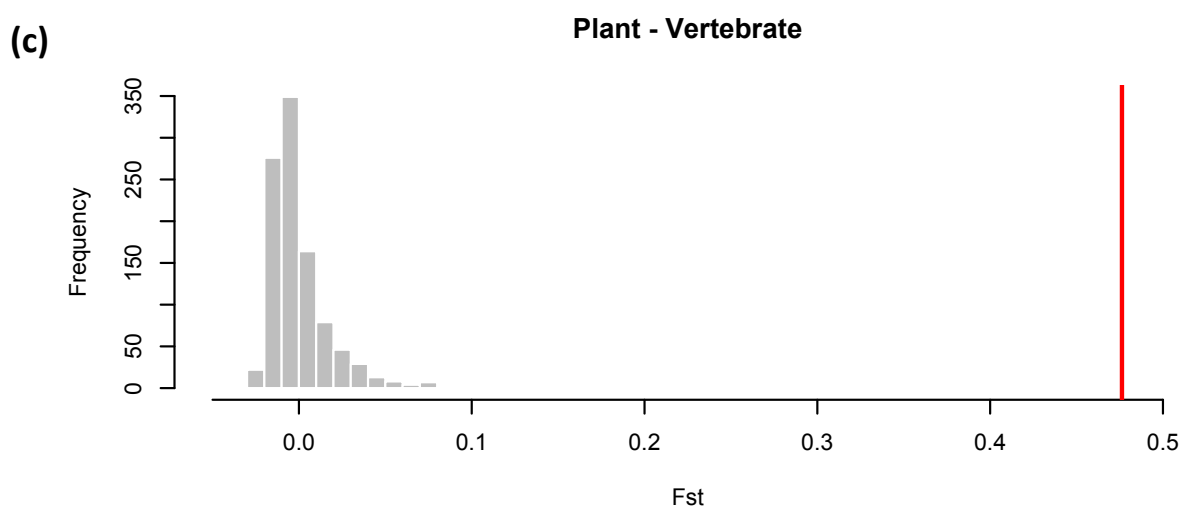

Supplementary Figure 2

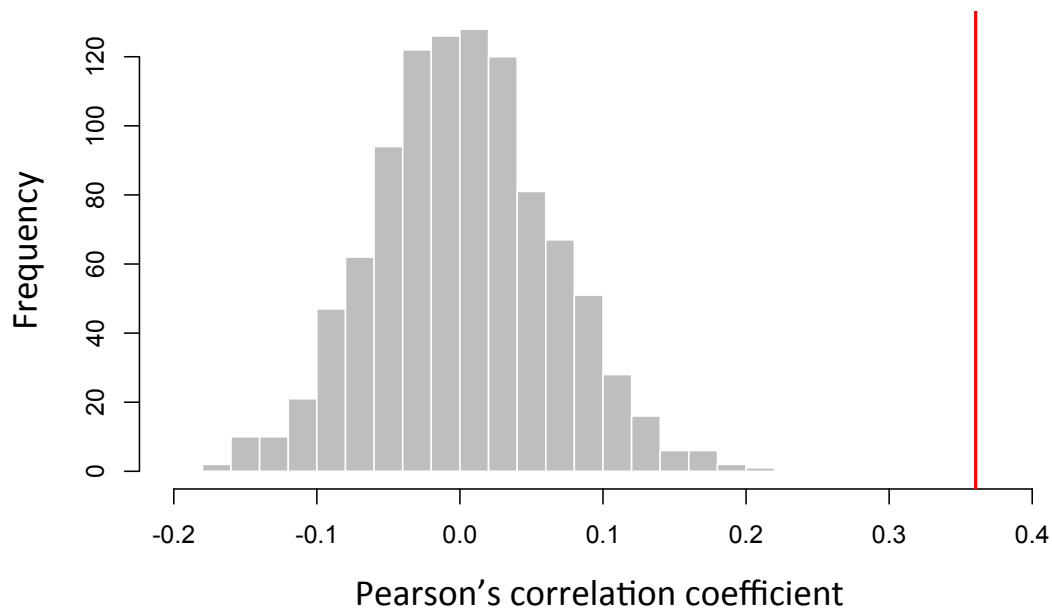

Supplementary Figure 3

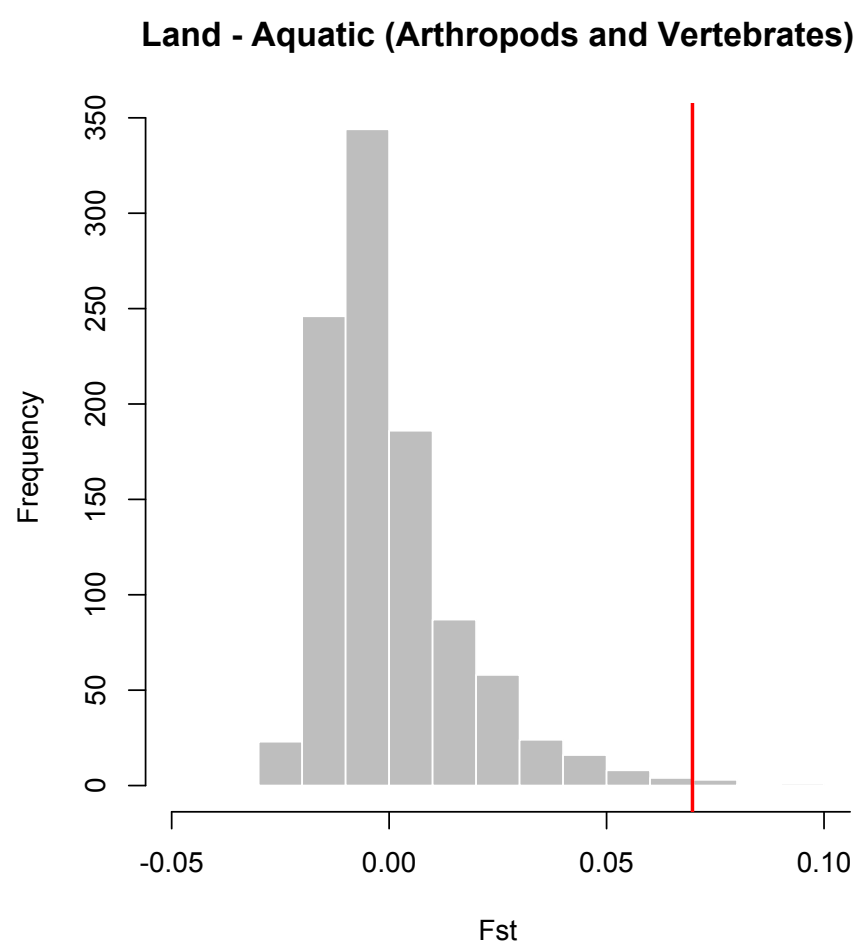

## Supplementary Figure 4

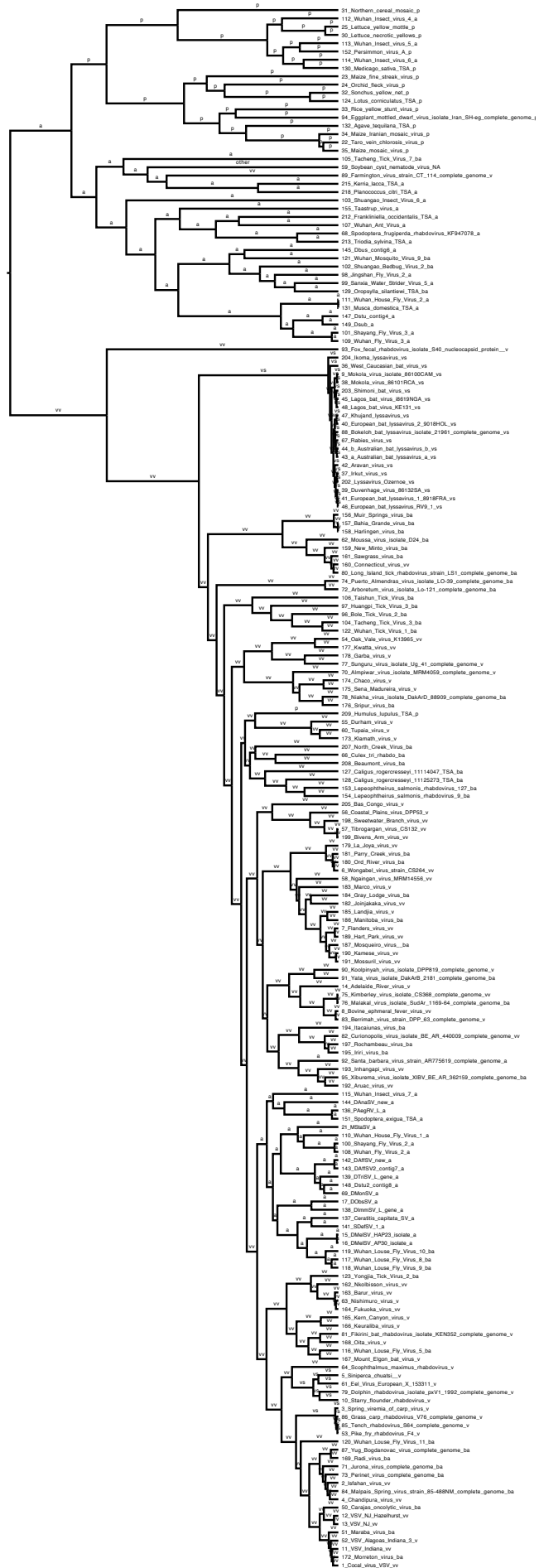

Supplement: Supplementary Data S1 [file b10ca1e197aa40e5c930139413c1ccc3_Supplementary_Figures_S1-S4.pdf]
